# Development and Characterization of Phage-Display-Derived Novel Human Monoclonal Antibodies against the Receptor Binding Domain of SARS-CoV-2

**DOI:** 10.3390/biomedicines10123274

**Published:** 2022-12-17

**Authors:** Ji Woong Kim, Sung Won Min, Jichul Lee, Ha Gyeong Shin, Hye Lim Choi, Ha Rim Yang, Ji Hyun Lee, Yea Bin Cho, Hyunbo Shim, Sukmook Lee

**Affiliations:** 1 Department of Chemistry, Kookmin University, Seoul 02707, Republic of Korea; 2 Research Center, SG Medical, Seoul 05548, Republic of Korea; 3 Department of Biopharmaceutical Chemistry, Kookmin University, Seoul 02707, Republic of Korea; 4 Department of Life Sciences, Ewha Womans University, Seoul 03760, Republic of Korea; 5 Biopharmaceutical Chemistry Major, School of Applied Chemistry, Kookmin University, Seoul 02707, Republic of Korea; 6 Antibody Research Institute, Kookmin University, Seoul 02707, Republic of Korea

**Keywords:** SARS-CoV-2, human antibody, neutralization, sandwich ELISA

## Abstract

Severe acute respiratory syndrome coronavirus-2 (SARS-CoV-2) has resulted in an ongoing global pandemic crisis, caused by the life-threatening illness coronavirus disease 2019 (COVID-19). Thus, the rapid development of monoclonal antibodies (mAbs) to cope with COVID-19 is urgently necessary. In this study, we used phage display to develop four human mAbs specific to the receptor-binding domain (RBD) of SARS-CoV-2. Our intensive in vitro functional analyses demonstrated that K102.1, an anti-SARS-CoV-2 RBD-specific mAb, exerted potent neutralizing activity against pseudoviral and live viral infection and the interaction between SARS-CoV-2 RBD and human angiotensin-converting enzyme 2. Monotherapy with K102.1 also revealed the therapeutic potential against SARS-CoV-2 infection in vivo. Further, this study developed a sandwich enzyme-linked immunosorbent assay with a non-competing mAb pair, K102.1 and K102.2, that accurately detected the RBDs of SARS-CoV-2 wild-type and variants with high sensitivity in the picomolar range. These findings suggest that the phage-display-based mAb selection from an established antibody library may be an effective strategy for the rapid development of mAbs against the constantly evolving SARS-CoV-2.

## 1. Introduction

Since the first outbreak in late 2019, coronavirus disease 2019 (COVID-19), caused by infection with severe acute respiratory syndrome coronavirus 2 (SARS-CoV-2), has been one of the greatest global human health threats in the 21st century [1]. Owing to its highly transmissible and rapidly mutating nature, SARS-CoV-2 leads to high rates of morbidity and mortality, which has resulted in an unprecedented socioeconomic crisis worldwide [2,3,4]. As of early September 2022, approximately 609 million people have been infected with COVID-19, with a confirmed death toll of 6.5 million individuals worldwide [5]. The symptoms of SARS-CoV-2 infection vary from mild disease to critical illness, including respiratory failure and death. The most common manifestations of COVID-19 are respiratory symptoms, such as fever, dry cough, and dyspnea [6,7]. However, severe cases of sepsis, secondary infections, and organ failure have also been reported [8,9,10].

SARS-CoV-2 is a single-stranded RNA virus that belongs to the beta-coronavirus family and exhibits 79% nucleotide identity with the sequence of SARS-CoV. Spike (S) proteins, composed of two subunits, S1 and S2, exist on the outer surface of the SARS-CoV-2 envelope [11]. Increasing evidence indicates that the receptor-binding domain (RBD; residues 331–524) of the S1 subunit is crucial for initiating viral entry by specifically interacting with human angiotensin-converting enzyme 2 (hACE2), a receptor on the surface of host cells [12,13]. For instance, a variety of interaction blockades, including antibodies, nanobodies, aptamers, peptides, and Fc-fusion proteins have been actively discovered and validate the importance of RBD-hACE2 interaction in SARS-CoV-2 infection [14,15,16,17,18,19,20,21,22]. Recent clinical trials of RBD-specific antibodies have also shown that RBD is a key therapeutic target in COVID-19 [23]. Furthermore, current vaccines targeting the RBD of the S protein of SARS-CoV-2 significantly increased the neutralization of antibodies to inhibit RBD-hACE2 interaction and prevent COVID-19 [24,25].

Monoclonal antibody (mAb) is a powerful tool for the treatment of chronic diseases, including cancers and acute infectious diseases, owing to its high specificity and efficacy [26,27]. Traditionally, mAbs are generated using consecutive processes of immunization and hybridoma technology, which is time consuming and labor intensive [28]. Furthermore, their immunogenicity risk also requires an additional humanization process for clinical use [29]. The recent advance of recombinant DNA technology has enabled the construction of human-naïve or synthetic antibody libraries, phage-display antibody selection, and overproduction of selected antigen-specific mAbs for academic and industrial applications [30,31]. However, the emergence of new SARS-CoV-2 variants with accumulated mutations have weakened the virus neutralization activity of existing RBD-specific mAbs [32]. Therefore, the rapid development of mAbs targeting the RBDs of an emerging SARS-CoV-2 variant will be important for timely measure and effective management against COVID-19.

The goal of this study is to employ biopanning with phage-display technology to rapidly isolate human antibodies from an established human antibody library, that specifically binds to the RBD of the SARS-CoV-2 S protein. Using intensive biochemical, molecular, and virological testing, we demonstrate the broad and potent neutralizing activity of the selected mAb with high affinity and specificity. Furthermore, based on a noncompeting antibody pair with K102.1 and K102.2, we generate and validate sandwich enzyme-linked immunosorbent assay (ELISA) that is highly sensitive and accurate for the detection of the RBDs of SARS-CoV-2. Taken together, these findings suggest that the phage-display-based mAb selection from an established antibody library may be useful for the rapid development of mAbs against the fast-evolving SARS-CoV-2.

## 2. Materials and Methods

### 2.1. Isolation of SARS-CoV-2 RBD-Specific Single-Chain Variable Fragments (scFvs) Using Phage-Display Technology

Biopanning was performed to isolate human antibodies specific to the SARS-CoV-2 RBD from a human synthetic scFv antibody library, as previously described [33]. Briefly, four rounds of biopanning were conducted to select SARS-CoV-2 RBD-specific scFv clones using Dynabeads™ M-270 epoxy magnetic beads (Invitrogen, Carlsbad, CA, USA) coated with 4 μg recombinant SARS-CoV-2 RBD (Cat#40592-V08B; Sino Biological, Beijing, China). Ninety-six clones were randomly selected from the output plate colonies and tested for reactivity to the SARS-CoV-2 RBD using phage ELISA.

### 2.2. DNA Cloning

DNA cloning was performed to convert the selected scFv clones into immunoglobulin G1 (IgG1) form. Each variable heavy and light chain gene of the scFv clones was individually amplified by polymerase chain reaction (PCR) using the following primers: forward and reverse primers for the heavy chains 5′-AGTGTGCT GGAATTCGCTGCCACTATGGAATGGAGCTGGGTCTTTCTCTTCTTCCTGTCAGTAACAACAGGTGTCCTTTCCGAGGTGCAGCTGTTGGAGTCTGGGG-3′, 5′-AAGACCGATGGGCCCTTGGTTGAGGCTGAGCTCACGGTGACCAGTGTACCC-3′; forward and reverse primers for light chains 5′-TGCAGCCACCGTACGTAGGACCGTCAGCTTG GTGCCTCCG-3′, 5′-GGGAGACCCAAGCTTGCCGCAACCATGGAGACACATTCCCA GGTCTTTGTATATATGTTGCTGTGGCTTTCAGGCGTTGAAGGGCAGTCTGTGCTGACTCAGCCACCCT-3′. The corresponding heavy chain and light chain PCR fragments were cloned into a mammalian expression pcDNA3.1 vector (Thermo Fisher Scientific, Waltham, MA, USA), encoding human IgG1.

To generate HA-tagged IgG1 mAbs, the HA-tag sequence (YPYDVPDYA) was incorporated into the C-terminus of the fragment crystallizable region of human IgG1. Each of the heavy and light chain variable region genes of the scFv clones were subcloned into the HA-tagged vector as described above.

### 2.3. Cell Culture

HEK293T cells were purchased from the American Type Culture Collection (ATCC, Rockville, MD, USA). VeroE6 cells were obtained from the Korea Microbial Resource Center (KCTC; Daejeon, Republic of Korea). Expi293^TM^ cells were obtained from Thermo Fisher Scientific. The HEK293T and VeroE6 cells were cultured in Dulbecco’s modified Eagle’s medium (Thermo Fisher Scientific), supplemented with 10% (*v*/*v*) fetal bovine serum (Thermo Fisher Scientific) and 1% (*v*/*v*) penicillin–streptomycin (Thermo Fisher Scientific) at 37 °C under an atmosphere with 5% CO_2_. The Expi293^TM^ cells were cultured in Expi293 expression medium (Gibco, Waltham, MA, USA) and maintained at 37 °C in an atmosphere of 8% CO_2_.

### 2.4. Production of SARS-CoV-2 RBD IgG mAbs

To produce SARS-CoV-2 RBD IgG mAbs, recombinant vectors that encode SARS-CoV-2 RBD antibodies were transfected into Expi293^TM^ cells using the Expi293 transfection kit (Thermo Fisher Scientific) following the manufacturer’s instructions. Seven days after transfection, the resulting supernatant was collected for antibody purification using affinity chromatography with Protein A Sepharose^®^ (Cytiva, Marlborough, MA, USA), as previously described [34].

### 2.5. Surface Plasmon Resonance (SPR) Binding Studies

Real-time measurement of kinetic binding between the SARS-CoV-2 RBD and selected mAbs was performed using the iMSPR-mini SPR instrument (Icluebio, Seongnam, Republic of Korea). The SARS-CoV-2 RBD (Cat#40592-V08B) was immobilized on a research-grade carboxylic acid (COOH) sensor chip (Icluebio) using an amine coupling kit (Icluebio) according to the manufacturer’s instructions. Next, increasing concentrations (8, 16, 32, 64, and 128 nM) of the selected mAbs in running buffer containing 10 mM HEPES-buffered saline (pH 7.4), 2 mM CaCl_2_, 1 mM MnCl_2_, 700 mM NaCl, and 0.005% (*v*/*v*) Tween 20 were injected at a flow rate of 50 µL/min at room temperature (RT). Each association phase occurred for 2 min and the dissociation phase occurred for 5 min. To regenerate the sensor chips after each cycle, 10 mM glycine-HCl (pH 2.5) was injected to remove bound antibodies from the chip surface.

To confirm the additional binding of human influenza hemagglutinin-tagged K102.2 (K102.2-HA) on the SARS-CoV-2 RBD-K102.1 complex, 128 nM K102.1 and K102.2-HA mAbs were sequentially applied to the immobilized SARS-CoV-2 RBD at RT with a flow rate of 50 µL/min. All SPR data were analyzed using the iMSPR analysis software (Icluebio).

### 2.6. Protein-Protein Interaction Inhibition Assay

The neutralization assay to examine the inhibitory effect of the selected mAbs on the interaction between the SARS-CoV-2 RBD and hACE2 was performed using ELISA. Briefly, 50 ng of purified Fc-tagged hACE2 (R&D Systems, Minneapolis, MN, USA) was coated onto each well of a 96-well plate for 2 h at RT. After washing with immuno-buffer (BPS Bioscience, San Diego, CA, USA), the wells were blocked with blocking buffer (BPS Bioscience) for 1 hr at RT. Simultaneously, 25 nM purified wild-type RBD histidine-tagged (RBD-His) (Cat#40592-V08B, Sino Biological), Alpha variant-RBD-His (Cat#40592-V08H82, Sino Biological), and Delta variant-RBD-His (Cat#40592-V08H90, Sino Biological) were pre-incubated in the absence or presence of increasing concentrations of mAbs (50, 16.67, 5.56, 1.85, 0.62, 0.21, 0.069, and 0.023 nM) for 1 hr at RT. After washing with immuno-buffer thrice, the pre-incubated mixtures were added to the wells for 1 hr at RT. Then, after washing with immuno-buffer thrice, horseradish peroxidase (HRP)-conjugated anti-His secondary Ab (BPS Bioscience) was added and incubated for 1 h at RT. The neutralization activity was detected using an enhanced chemiluminescence substrate (BPS Bioscience), and chemiluminescence intensity was measured using a Synergy H1 microplate reader (BioTek Instruments, Winooski, VT, USA). Nonlinear regression curves were analyzed using the Prism 8 (GraphPad software, La Jolla, CA, USA) to calculate half-maximal inhibition concentration (IC_50_) values.

### 2.7. In Vitro SARS-CoV-2 Pseudovirus Neutralization Assay

Pseudotyped, replication-deficient, Moloney murine leukemia virus particles carrying the S protein of wild-type (Cat#SCV2-PsV-001), B.1.1.7 (Cat#SCV2-PsV-UK; Alpha), and B.1.617.2 (Cat#SCV-PsV-Delta; Delta) variant SARS-CoV-2 and expressing a firefly luciferase reporter gene were obtained from eEnzyme (Gaithersburg, MD, USA). To determine the neutralization activity of the selected mAbs against pseudovirus infection, 1 × 10^4^ stable hACE2-overexpressing 293T cells (hACE2/293T) were seeded in 96-well tissue culture plates and incubated overnight. Serially diluted mAbs (50, 16.67, 5.56, 1.85, 0.62, 0.21, 0.069, and 0.023 nM) were pre-incubated at RT for 10 min with 50 μL pseudovirus (1 × 10^7^ PFU/mL) added to the cells, and the plates were incubated for 24 hr. Firefly luciferase reporter gene expression (indicative of viral presence) was measured using ONE-Glo^TM^ luciferase substrate (Promega, Madison, WI, USA). Briefly, the culture medium was removed and incubated with 100 μL ONE-Glo^TM^ substrate. After 5 min, 70 μL supernatant was transferred to a white, flat-bottom 96-well assay plate (Corning, Lowell, MA, USA), and luminescence was measured using a microplate reader. The obtained relative luminescence units were normalized to those derived from cells infected with SARS-CoV-2 pseudovirus in the absence of mAbs. IC_50_ values were determined using four-parameter nonlinear regression (GraphPad software).

### 2.8. In Vitro SARS-CoV-2 Live Virus Neutralization Assay

SARS-CoV-2 (BetaCoV/korea/KCDC03/2020, NCCP no. 43326) was provided by the Korea Disease Control and Prevention Agency (KDCA, Osong, Republic of Korea). The experiment was performed in the biosafety level 3 laboratory (BSL3) facility of Korea Zoonosis Research Institute at Jeonbuk National University. The virus was prepared by propagation in Vero E6 cells, titered using a plaque assay, and stored at −80 °C for in vitro infection assessment. A total of 1.5 × 10^4^ VeroE6 cells in 100 μL of culture medium were seeded onto 96-well tissue-culture plates and incubated overnight. Serial dilutions of mAbs (50 mL) were pre-incubated with 50 μL SARS-CoV-2 (4 × 10^2^ TCID_50_/mL) for 1 hr at RT. The medium was then removed from the seeded VeroE6 cells and replaced with the virus/antibody mixture. Four days post-inoculation, the cytopathic effect (CPE) was monitored, and neutralization potency was determined by viral RNA quantification using quantitative reverse transcription polymerase chain reaction (RT-qPCR) with the CFX96 Real-Time PCR Detection System (Bio-Rad Laboratories, Hercules, CA, USA). Reverse transcription of the total RNA was conducted using the High-Capacity cDNA Reverse Transcription Kit (Applied Biosystems, Foster, CA, USA). The reaction mixture (20 μL total) contained 2 μL template cDNA, 10 μL 2× Premix Ex Taq, 200 nM primer, and probe. The following primers were used: Nucleocapsid (N) gene: forward primer 5′-GACCCCAAAATCAGCGAAAT-3′, reverse primer 5′-TCTGGTTACTGCCAGTTGAAT CTG-3′, probe 5′-ACCCCGCATTACGTTTGGTGGACC-3′; Envelope (E) gene: forward primer 5′-ACAGGTACGTTAATAGTTAATAGCGT-3′, reverse primer 5′-ATATTGCAGC AGTACGCACACA-3′, probe 5′-ACACTAGCCATCCTTACTGCGCTTCG-3′; RNA-dependent RNA polymerase (RdRp) gene: forward primer 5′-ATGAGCTTAGTCC TGTTG-3′, reverse primer 5′-CTCCCTTTGTTGTGTTGT-3′, probe 5′-AGATGTCTTGTGCTGCCGGTA-3′. The thermal cycle conditions included 2 min at 50 °C, 10 min at 92 °C, followed by 30 cycles of 15 s at 92 °C, and 1 min at 60 °C.

### 2.9. In Vivo Mouse Study

For in vivo efficacy testing, 8-week-old female hACE-2 transgenic (TG) mice (B6.Cg-Tg (K18-ACE2)2Prlmn/J; The Jackson Laboratory, Sacramento, CA, USA) were housed in a certified animal BSL3 (ABSL3) facility (Korea Zoonosis Research Institute, Iksan, Republic of Korea). All procedures were approved by the Institutional Animal Care and Use Committee (IACUC) at KOTUS (No. 22-KE-0076), and all experimental protocols requiring biosafety were approved by the Institutional Biosafety Committee of Jeonbuk National University (approval number: JBNU 2020-11-003-003). The experiment was performed in a biosafety cabinet at the BSL3 and ABL3 facilities of Korea Zoonosis Research Institute at Jeonbuk National University. The hACE2-transgenic (hACE2-TG) mice (*n* = 5) were intranasally inoculated with 30 μL of wild-type SARS-CoV-2 (1 × 10^4^ PFU) under anesthesia. Three hours post infection, mAbs in PBS were injected intravenously.

The SARS-CoV-2 viral load in lung tissues was determined by using RT-qPCR. Lung tissues were harvested from hACE2-TG mice at 5 days after wild-type SARS-CoV-2 infection, and total RNAs were extracted from the collected tissues using Wizol^TM^ Reagent (Wizbiosolutions, Seongnam, Republic of Korea). Samples were subjected to reverse transcription-quantitative polymerase chain reaction (RT-qPCR) using a CFX96 Real-Time PCR Detection System (Bio-Rad Laboratories). Following reverse transcription of total RNA using a High-Capacity cDNA Reverse Transcription Kit (Applied Biosystems, Foster, CA, USA), the reaction mixture (20 μL total) contained 2 μL of template cDNA, 10 μL of Premix Ex Taq, 200 nM primer, and a probe (E gene: forward primer 5′-ACAGGTACGTTAATAGTTAATAGCGT-3′, reverse primer 5′-ATATTGCAGC AGTACGCACACA-3′, probe 5′-ACACTAGCCATCCTTACTGCGCTTCG-3′; RdRp gene: forward primer 5′-ATGAGCTTAGTCCTGTTG-3′, reverse primer 5′-CTCCCTTTGTTGT GTTGT-3′, probe 5′-AGATGTCTTGTGCTGCCGGTA-3′). These reaction mixtures were denatured at 95 °C for 30 s, and then subjected to 45 cycles of 95 °C for 5 s and 60 °C for 20 s. After completion of the reaction cycles, the temperature was increased from 65 to 95 °C at a rate of 0.2 °C/15 s and fluorescence was measured every 5 s to construct a melting curve. A control sample lacking the template DNA was run with each assay. The authenticity of the amplified product was determined using melting curve analysis. All data were analyzed using Bio-Rad CFX Manager analysis software version 2.1 (Bio-Rad Laboratories). The viral load was expressed by the copy number of viral RNA per nanogram of total RNA.

### 2.10. Histology

Excised mouse lung tissues were fixed using 4% (*v*/*v*) paraformaldehyde (PFA) in PBS and processed for paraffin embedding. The paraffin blocks were cut to 3 µm thickness using a microtome (HistoCore MULTICUT R; Leica, Germany) and mounted on silane-coated glass slides (5116-20F; Muto, Tokyo, Japan). Hematoxylin and eosin, periodic acid–Schiff, and modified Masson’s trichrome stains were used to identify histopathological changes in all the organs. The histopathology of the lung tissue was observed using light microscopy (Axio Scope A1; Carl Zeiss, Jena, Germany). Pathological scores were determined based on the percentage of inflammation area for each section in each group using the following scoring system: 0, no pathological change; 1, affected area (≤10%); 2, affected area (10–50%); 3, affected area (≥50%); an additional 0.5 was added when pulmonary edema and/or alveolar hemorrhage was observed.

### 2.11. Sandwich ELISA

Ninety-six-well high-binding microplates (Corning) coated with a capture antibody were blocked using 3% (*w*/*v*) bovine serum albumin (BSA) in phosphate-buffered saline (PBS) for 2 hr at 37 °C. Next, 100 μL of increasing concentrations of the RBDs of wild-type SARS-CoV-2 or mutants (A435S; Sino#40592-V08H4, G476S; Sino#40592-V08H8, F342L; Sino#40592-V08H6, N354D; Sino#40592-V08H2, V483A; Sino#40592-V08H5, V341I; Sino#40592-V08H11, B.1.1.7; Sino#40592-V08H82, and B.1.617.2; Sino#40592-V08H90) were added to the wells, and the microplate was incubated for 2 hr at 37 °C. The plates were washed thrice with PBS containing 0.05% (*v*/*v*) Tween 20 (PBST) and incubated with HRP-conjugated anti-HA antibody (1:3000, Bethyl Laboratories, Montgomery, TX, USA) for 1 hr at 37 °C. Following three washes with PBST, 3,3′,5,5′-tetramethylbenzidine substrate solution (Thermo Fisher Scientific) was added to each well and allowed to react with HRP for 5 min. The reaction was terminated by adding 100 μL of 1 M H_2_SO_4_. The absorbance of each sample was measured at 450 nm using a microplate reader.

### 2.12. Statistical Validation of Sandwich ELISA

Intra-assay precision was determined by measuring samples run six times in triplicate within the same assay run. Inter-assay precision was determined by measuring a sample in triplicate in six separate assay runs. The mean and standard deviation (SD) was calculated. The coefficient of variation (CV) was calculated as follows: CV (%) = (SD/mean) × 100. Recovery was calculated as follows: [average measured concentration/expected concentration] × 100.

## 3. Results

### 3.1. Isolation and Biochemical Characterization of SARS-CoV-2 RBD-Specific mAbs

**Figure 1:**
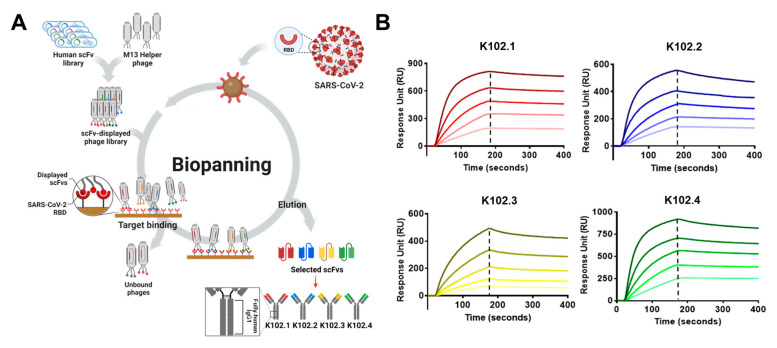
Isolation and characterization of SARS-CoV-2 RBD-specific mAbs. (**A**) Following biopanning using phage-display technology with wild-type SARS-CoV-2 RBD-coupled magnetic beads, four scFv clones specific to SARS-CoV-2 RBD were selected from the human synthetic antibody library and converted to generate IgG mAbs (K101.1–4). (**B**) Following overproduction and purification, the dissociation equilibrium constants of the selected mAbs were individually measured using surface plasmon resonance. The data are representative of two independent experiments.

To isolate novel anti-SARS-CoV-2 RBD-specific mAbs, biopanning was performed using phage-display technology from a human synthetic scFv library and four SARS-CoV-2 RBD-specific scFv clones were selected. Phage ELISA revealed that the selected scFv clones strongly bound to wild-type SARS-CoV-2 RBD antigen, but not to BSA as the negative control, and the clones had different complementarity-determining region sequences via DNA sequencing. Each scFv clone was converted to generate an IgG1 mAb. Following overproduction and purification, the resulting IgG mAbs were verified to have >90% purity by sodium dodecyl sulfate polyacrylamide gel electrophoresis and Coomassie Brilliant Blue staining. The selected IgG mAbs were designated K102.1, K102.2, K102.3, and K102.4 (Figure 1A) and were confirmed to specifically bind to the SARS-CoV-2 RBD, but not to BSA.

To determine the binding affinity of the selected IgG mAbs to wild-type SARS-CoV-2 RBD, real-time kinetics analysis was carried out. The SPR analysis results revealed that the equilibrium dissociation constants (K_D_) for K102.1, K102.2, K102.3, and K102.4 against the SARS-CoV-2 RBD were approximately 1.1, 2.5, 11.3, and 3.2 nM, respectively (Figure 1B and Table 1).

### 3.2. Neutralization of Selected SARS-CoV-2 RBD-Specific mAbs against SARS-CoV-2

**Figure 2:**
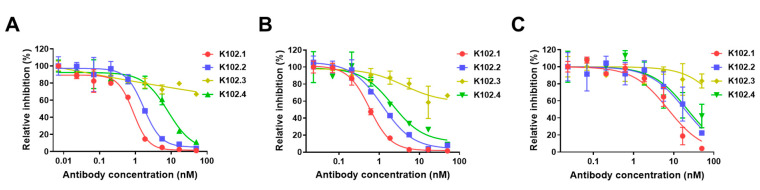
Concentration-dependent neutralizing effect of selected mAbs against interactions between hACE2 and RBDs of SARS-CoV-2. Interactions between recombinant hACE2 and RBDs of wild-type (**A**), Alpha (**B**), or Delta (**C**) variant SARS-CoV-2 were determined in the presence or absence of increasing concentrations of K102.1 (red), K102.2 (blue), K102.3 (yellow), and K102.4 (green). All values represent the mean ± SD of duplicate measurements from one of two independent experiments. The IC_50_ values were determined by log (inhibitor) response of nonlinear regression.

To assess the neutralizing ability of the selected IgG mAbs, ELISA-based neutralization assays were performed, wherein recombinant RBDs of wild-type and variant SARS-CoV-2 were incubated with recombinant hACE2 in the presence or absence of the selected IgG mAbs. K102.1 showed the most potent neutralizing activity, displaying the lowest IC_50_ values against interactions between hACE2 and RBDs of wild-type (0.9 nM), Alpha (7.27 nM), and Delta (1.6 nM) variant SARS-CoV-2 (Figure 2A–C and Table 2).

**Figure 3:**
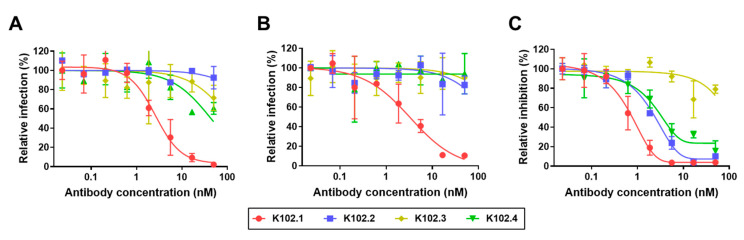
Concentration-dependent neutralizing effect of selected mAbs against pseudoviral SARS-CoV-2 infection. Neutralizing activity of K102.1 (red), K102.2 (blue), K102.3 (yellow), and K102.4 (green) against infection with pseudoviral wild-type (**A**), Alpha (**B**), and Delta (**C**) variant SARS-CoV-2. Values represent the mean ± SD of duplicate measurements from one of two independent experiments. The IC_50_ values were determined by log (inhibitor) response of nonlinear regression.

The neutralization activity of the selected IgG mAbs against pseudoviral SARS-CoV-2 infection was evaluated using stable hACE2-overexpressing 293T cells. Pseudotyped viruses are chimeric virions that consist of surrogate viral cores with viral S proteins at their surface and are the most common tool for studying the neutralizing effect of tested antibodies on viral entry. K102.1 had the strongest inhibitory effect against the entry of pseudoviral wild-type, Alpha, and Delta variant SARS-CoV-2 with IC_50_ values of approximately 2.6, 6.4, and 3.3 nM, respectively (Figure 3A–C).

**Figure 4:**
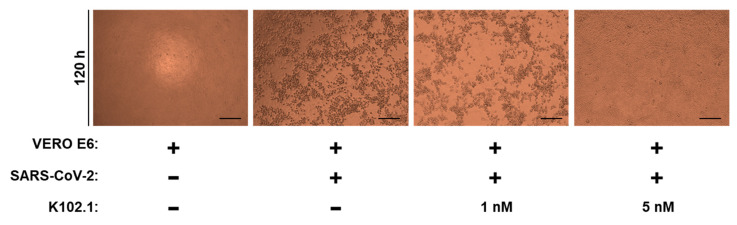
Neutralizing effect of selected mAb against live SARS-CoV-2 infection. Wild-type SARS-CoV-2-induced CPE on Vero E6 cells was observed in the absence or presence of indicated concentrations of K102.1 at 120 h using bright-filed microscopy. Scale bars: 200 μm.

To investigate the neutralizing effect of K102.1 against live SARS-CoV-2 infection, CPE reduction assays were performed following incubation of Vero E6 cells with wild-type SARS-CoV-2 in the absence or presence of K102.1. The results revealed that the CPE of SARS-CoV-2 was partially inhibited by 1 nM K102.1 and completely inhibited by 5 nM K102.1 (Figure 4). RT-qPCR analysis was conducted to further investigate the effect of K102.1 on the expression of SARS-CoV-2 viral genes associated with N, E protein, and RdRp. Concurring with the results shown in Figure 4, 5 nM K102.1 almost completely inhibited the expression of all tested viral genes (Supplementary Figure S3).

### 3.3. In Vivo Efficacy Evaluation of K102.1 in Wild-Type SARS-CoV-2-Infected Animal Model

**Figure 5:**
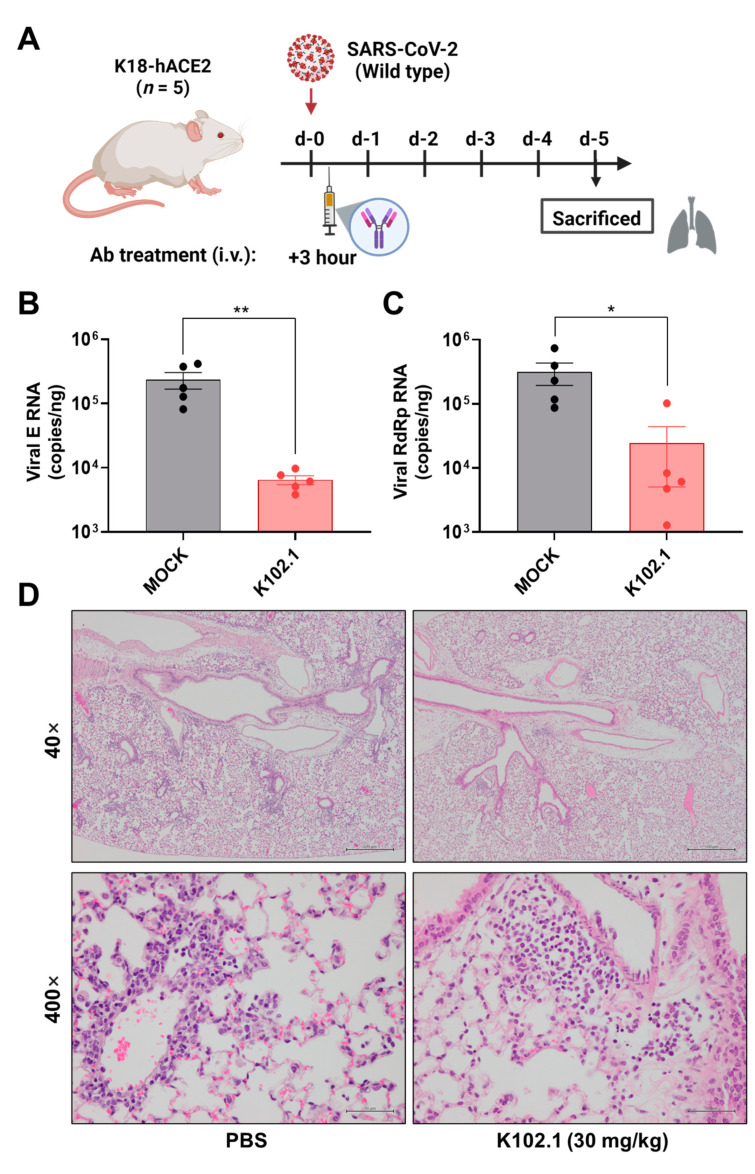
In vivo efficacy evaluation of K102.1 in hACE2 transgenic mice infected with wild-type SARS-CoV-2. (**A**) Schematic description of the experimental design for in vivo efficacy evaluation of K102.1 in wild-type SARS-CoV-2-infected K18-hACE2 transgenic mice (*n* = 5). The level of the viral E (**B**) or RdRp (**C**) gene expression in lung tissue was measured using RT-qPCR at 5 dpi. Data were statistically analyzed using two-tailed Student’s *t*-test (* *p* < 0.05, ** *p*  < 0.01). (**D**) Two representative histopathological images of the lung tissue in each group at 5 dpi. Scale bar (40×): 500 µm; scale bar (400×): 50 µm. All values represent the mean ± SD of five biological replicates.

To evaluate the in vivo efficacy of K102.1 against wild-type SARS-CoV-2, the viruses were intranasally administered in K18-hACE2 TG mice. After 3 h of infection, 30 mg/kg of K102.1 was intravenously injected to the mice (Figure 5A). All mice were sacrificed at 5 days post infection (dpi) and lung samples were subjected to RT-qPCR to determine the relative expression of viral E and RdRp genes. The result shows that the expression of both viral genes significantly reduced in K102.1-treated group when compared to the PBS-treated group (Figure 5B,C).

Pathological examination of the lungs from wild-type SARS-CoV-2 infected mice at 5 dpi showed that PBS-treated mice showed a pathological score of ≥ 1 with significant pulmonary lesions. In comparison, all the K102.1-treated mice showed a pathological score of ≤ 1 (Supplementary Table S1). Further histopathological analyses revealed normal features in the K102.1-treated lungs, whereas PBS-treated mice exhibited relatively severe pulmonary edema or alveolar hemorrhage (Figure 5D). Taken together, these results suggested that K102.1 may have excellent therapeutic potential against wild-type SARS-CoV-2 infection.

### 3.4. Identification of SARS-CoV-2 RBD-Specific mAb Pair for Sandwich ELISA

**Figure 6:**
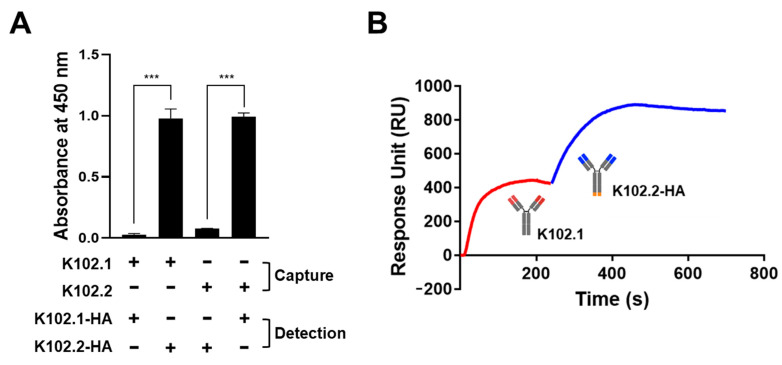
Identification of SARS-CoV-2 RBD-specific monoclonal antibody pair for sandwich ELISA. (**A**) Sandwich ELISA was conducted with untagged forms of K102.1 or K102.2 as capture antibodies and HA-tagged K102.1 (K102.1-HA) or K102.2 (K102.2-HA) as detection antibodies. Values represent the mean ± SD of duplicate measurements from one of two independent experiments. Data were statistically analyzed using two-tailed Student’s t-test (*** *p* < 0.001). (**B**) Additional binding of K102.2-HA (blue) to wild-type SARS-CoV-2 RBD after K102.1 (red) was saturated, as measured by surface plasmon resonance. The curves are representative of two independent experiments.

To identify whether K102.1 and K102.2, the mAbs with the highest affinity for wild-type SARS-CoV-2 RBD, would provide a suitable antibody pair for sandwich ELISA, hemagglutin (HA)-tagged K102.1 or K102.2 (K102.1-HA and K102.2-HA) were first generated by introducing HA tags at the C-terminus of the heavy chain of the mAb. Next, untagged forms of K102.1 or K102.2 immobilized as capture antibodies onto a microtiter plate were incubated with wild-type SARS-CoV-2 RBD in the presence or absence of K102.1-HA or K102.2-HA as detection antibodies. The results indicated that both the K102.1 and K102.2-HA pair and the K102.2 and K102.1-HA pair were suitable for sandwich ELISA (Figure 6A).

Competition assays were performed using SPR to further confirm the presence of independent binding of the selected mAb pair to wild-type SARS-CoV-2 RBD. Following saturation with K102.1 on the SARS-CoV-2 RBD-immobilized sensor chip, K102.B-HA was applied. The results indicated that K102.2-HA exhibited additional binding even after saturation of the SARS-CoV-2 RBD with K102.1, suggesting independent binding between the selected mAb pair and the SARS-CoV-2 RBD (Figure 6B).

### 3.5. Development and Characterization of Sandwich ELISA for Detection of Wild-Type SARS-CoV-2 RBD

**Figure 7:**
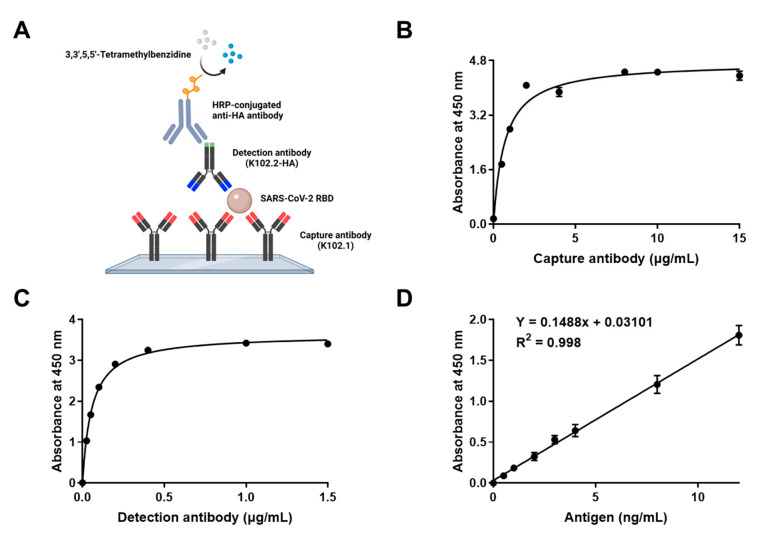
Generation and characterization of SARS-CoV-2 RBD sandwich ELISA. (**A**) Schematic representation of sandwich ELISA. (**B**) Optimizing the capture antibody (K102.1) concentration by sandwich ELISA. Microplate wells were coated with the indicated concentrations of K102.1, followed by incubation of the plates with 0.1 mg RBD of wild-type SARS-CoV-2 (antigen) and 2.5 μg/mL K102.2-HA (detection antibody). (**C**) Microplate wells were coated with 5 μg/mL K102.1, followed by incubation with 0.1 μg RBD of wild-type SARS-CoV-2. Titration of the indicated concentrations of K102.2-HA (detection) antibody was evaluated by sandwich ELISA. (**D**) The wild-type SARS-CoV-2 RBD calibration curve was constructed using 5 μg/mL capture antibody (K102.1) and 1 μg/mL detection antibody (K102.2-HA) to determine the limit of detection. All values represent the mean ± SD of triplicate measurements from six independent experiments.

Sandwich ELISA is a reliable and rapid detection tool for infectious viral antigens [35,36]. To establish a sandwich ELISA specific to wild-type SARS-CoV-2 RBD antigen, K102.1 was used as the capture antibody whereas K102.2-HA was used as the detection antibody. Specific recognition of the SARS-CoV-2 RBD antigen by the selected mAb pair was detected using chemiluminescence ELISA (Figure 7A).

Optimization of capture and detection antibody concentrations is a key factor in determining the sensitivity and working range of a sandwich ELISA system [37]. Various concentrations of capture and detection mAbs were applied to a microplate-based sandwich format of SARS-CoV-2 RBD ELISA. The results showed that the optimal concentration of the capture mAb (K102.1) was 5 μg/mL, and that of the detection mAb (K102.2-HA) was 1 μg/mL (Figure 7B,C).

The linear dynamic range of the calibration curve was determined to be between 0 and 12 ng/mL (equivalent to 0–480 pM) with the SARS-CoV-2 RBD. The reproducibility of the calibration curve was demonstrated using six independent assays. The SARS-CoV-2 RBD limit of detection (LOD) was estimated to be 0.8 ng/mL (equivalent to 32 pM) (Figure 7D). Next, intra-and inter-assay CVs and recoveries of the optimized sandwich ELISA were calculated. The intra- and inter-assay CVs for 5 ng/mL SARS-CoV-2 RBD were 8.46% and 9.52%, respectively. The intra- and inter-assay recoveries for 5 ng/mL SARS-CoV-2 RBD were 105.57% and 98.56%, respectively (Table 3). Intra- and inter-assay variations were considered acceptable (<10%), suggesting that the sandwich ELISA system provided a sensitive, accurate, and reliable technique for detecting wild-type SARS-CoV-2 RBD. Furthermore, to evaluate the utility of the developed sandwich ELISA against SARS-CoV-2 variants, the assay was performed in the presence of increasing concentrations (32 pM [LOD for wild-type SARS-CoV-2 RBD], 96 pM, or 200 pM) of SARS-CoV-2 RBDs with mutations, including A435S, G476S, F342L, N354D, V483A, V341I, N501Y (B.1.1.7; Alpha), and L452R/T478K (B.1.617.2; Delta). The results demonstrated that the developed sandwich ELISA system was highly sensitive to all eight SARS-CoV-2 RBD mutants in the picomolar range (Supplementary Figure S4).

## 4. Discussion

The rapid spread of highly transmissible SARS-CoV-2 has resulted in an unprecedented threat to public health and global socioeconomic crisis [38]. Since the COVID-19 outbreak, much attention has been paid to the development of a variety of mAb-based therapeutic interventions and detection tools for SARS-CoV-2 [39]. However, most of the existing SARS-CoV-2-specific mAbs have been derived from B cells of convalescent whole blood or immunized transgenic mice, which is highly time consuming and labor intensive [40,41,42]. This has been a major hurdle for implementing timely measures against a life-threatening COVID-19. Therefore, the rapid development of mAbs against emerging new SARS-CoV-2 variants is urgently required for the effective management of the COVID-19 pandemic. In the present study, four SARS-CoV-2 RBD-specific human mAbs were selected and generated using phage-display technology from an established human synthetic antibody library. Intensive characterization and functional studies demonstrated that K102.1 had potent neutralizing activity against wild-type, Alpha, and Delta variant SARS-CoV-2. Furthermore, the developed sandwich ELISA with K102.1 and K102.2, a non-competing mAb pair, was highly sensitive for detecting RBDs of wild-type and variant SARS-CoV-2. The study findings not only present the potential use of phage-display-derived mAbs that we developed, but also provide insights for the rapid development of mAbs against the fast-evolving SARS-CoV-2.

The RBD of the SARS-CoV-2 S protein directly interacts with hACE2, which is the primary host cell receptor needed for viral entry [43]. Many studies have shown that this interaction is a key target for the development of mAb-based therapeutics against COVID-19 [44,45,46,47,48,49]. The results of the present study provide several items of evidence that K102.1, a phage display-derived mAb, may have therapeutic potential against SARS-CoV-2. For example, K102.1 is a fully human mAb isolated from a human synthetic antibody library, thereby inducing a lower probability of immunogenicity risk. Moreover, it specifically and strongly binds to the SARS-CoV-2 RBD with high binding affinity (approximately 1 nM). The results of the ELISA-based neutralization assays revealed that K102.1 specifically inhibited interactions between hACE2 and RBDs of wild-type, Alpha, and Delta variant SARS-CoV-2. Furthermore, K102.1 exerted excellent neutralization effects (nanomolar IC_50_) against infection with SARS-CoV-2 pseudoviruses, including wild-type, Alpha, and Delta variants. In addition, neutralization assays with live virus revealed that 5 nM K102.1 almost completely inhibited both CPE and viral gene expression of SARS-CoV-2. Finally, through intravenous injections, the most widely used clinical route of antibody drug administration for systemic circulation [50], monotherapy with 30 mg/kg of K102.1 showed a significant neutralizing ability against wild-type SARS-CoV-2 infected-K18-hACE2 TG mice in vivo.

A rapid and accurate detection for SARS-CoV-2 in serum or plasma is important to quantify the number and severity of patients infected with SARS-CoV-2, and to predict the efficacy of drugs following drug administration [51]. In the present study, a sandwich ELISA assay was developed with a non-competing pair of anti-SARS-CoV-2 RBD-specific mAbs, K102.1 and K102.2, as the capture and detection antibodies. The assay was accurate and highly sensitive for detecting the RBDs of wild-type and variant SARS-CoV-2. The developed sandwich ELISA had an LOD for wild-type SARS-CoV-2 RBD in the picomolar range (32 pM) and demonstrated a wide dynamic range. Most previously developed sandwich ELISA systems to detect viral antigens of SARS-CoV-2 are in nano- to picomolar ranges, which is comparable to the results of our immunoassay. For example, Svobodova et al. and Dominico et al. reported the LOD values of the sandwich ELISA as ~270 pM and ~1 nM, respectively. The intra-and inter-assay CVs supported the accuracy and reliability of the SARS-CoV-2 RBD detection sandwich ELISA. Further analyses confirmed that the assay could detect the RBDs of SARS-CoV-2 with A435S, N354D, G476S, V483A, F342L, N501Y (Alpha), and L452R/L484K (Delta) mutations in the picomolar range (≥96 pM).

## 5. Conclusions

The present study demonstrated that the phage-display-derived novel mAbs used in this study effectively neutralized SARS-CoV-2 infection, while providing accurate and highly sensitive SARS-CoV-2 detection. The results suggest that the phage-display-based mAb selection from an established antibody library may be an effective strategy for the rapid development of mAbs against the fast-evolving SARS-CoV-2. Future research will employ this approach to rapidly develop various mAbs specific to the emerging SARS-CoV-2 variants, such as Omicron, and further validate a wide range of their utility against COVID-19.

## Figures and Tables

**Table 1:** Evaluation of binding kinetics of selected antibodies to wild-type SARS-CoV-2 RBD.

Antibody	K_D_ (nM)	K_a_ (1/M^−1^ s^−1^)	K_d_ (s^−1^)
K102.1	1.11	5.95 × 10^5^	6.61 × 10^−4^
K102.2	2.45	4.03 × 10^5^	9.89 × 10^−4^
K102.3	11.3	3.43 × 10^4^	3.89 × 10^−4^
K102.4	3.16	3.15 × 10^5^	9.96 × 10^−4^

K_D_, equilibrium dissociation constant; K_a_, association constant; K_d_, dissociation constant.

**Table 2:** Neutralizing effect of selected mAbs on direct interaction between hACE2 and RBDs of SARS-CoV-2 wild-type and variants.

RBD Type	IC_50_ (nM)			
	K102.1	K102.2	K102.3	K102.4
Wild-type	0.89 ± 0.15	1.71 ± 0.23	ND	8.15 ± 0.82
B.1.1.7 (Alpha)	7.27 ± 1.01	18.85 ± 2.41	ND	21.83 ± 2.55
B.1.617.2 (Delta)	1.57 ± 0.52	2.99 ± 0.51	ND	12.54 ± 1.42

ND, not determined.

**Table 3:** Reliability and recovery of developed sandwich ELISA for wild-type SARS-CoV-2 RBD.

Spiked Level (ng/mL)	Intra-Assay (*n* = 6)			Inter-Assay (*n* = 6)		
	Mean ± SD (ng/mL)	Recovery (%)	CV (%)	Mean ± SD (ng/mL)	Recovery (%)	CV (%)
5	5.28 ± 0.45	105.57	8.46	4.93 ± 0.47	98.56	9.52

SD*,* standard deviation*;* CV*,* coefficient of variation.

## Data Availability

The data collected in this study are available from the corresponding author upon request.

## Supplementary Materials

The following supporting information can be downloaded at: https://www.mdpi.com/article/10.3390/biomedicines10123274/s1, Figure S1: Neutralizing effect of selected mAb against wild-type SARS-CoV-2 live viral infection in vitro.; Figure S2: Detection of multiple SARS-CoV-2 RBD antigens using developed sandwich ELISA.; Table S1: Histopathological evaluation and scoring of lungs from the SARS-CoV-2-infected K18-hACE2 mice model.
