# Supplementary material for: Development and Characterization of Phage-Display-Derived Novel Human Monoclonal Antibodies against the Receptor Binding Domain of SARS-CoV-2
Source: Biomedicines. 2022 Dec 17;10(12):3274. doi: 10.3390/biomedicines10123274 (PMC9775448; doi:10.3390/biomedicines10123274)
Supplement: Supplementary file 1 [file biomedicines-10-03274-s001.zip › biomedicines-2081109-supplementary.pdf]

Supplementary Table S1. Histopathological evaluation and scoring of lungs from the SARS-CoV-2-infected K18-hACE2 mice model.

Pathological score	Number of specimen (n = 5)	
	PBS	K102.1 (30 mg/kg)
0	0	1 (20%)
0.5	0	2 (40%)
1	2 (40%)	2 (40%)
1.5	1(20%)	0
2	2 (40%)	0
Mean*	1.5	0.6

*Mean = (pathological score × numbers of specimen)/total numbers of specimen

Pathological score = (0, 0%; 1, ≤10%; 2, 10%-50%; 3, ≥50%; +0.5, pulmonary edema or alveolar hemorrhage)

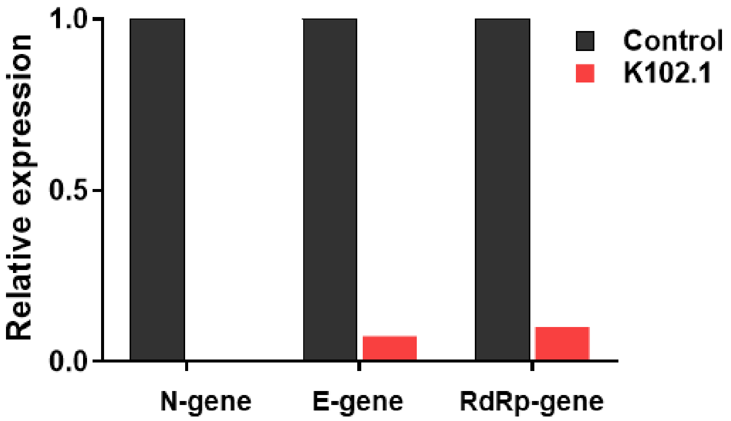

Supplementary figure 1. Neutralizing effect of selected mAb against wild-type SARS-CoV-2 live viral infection *in vitro*. Relative expression of the N, E, and RdRp gene was individually quantified using RT-qPCR in the absence (black) or presence (red) of 5 nM K102.1 at 120 h after wild-type SARS-CoV-2 infection in Vero E6 cells.

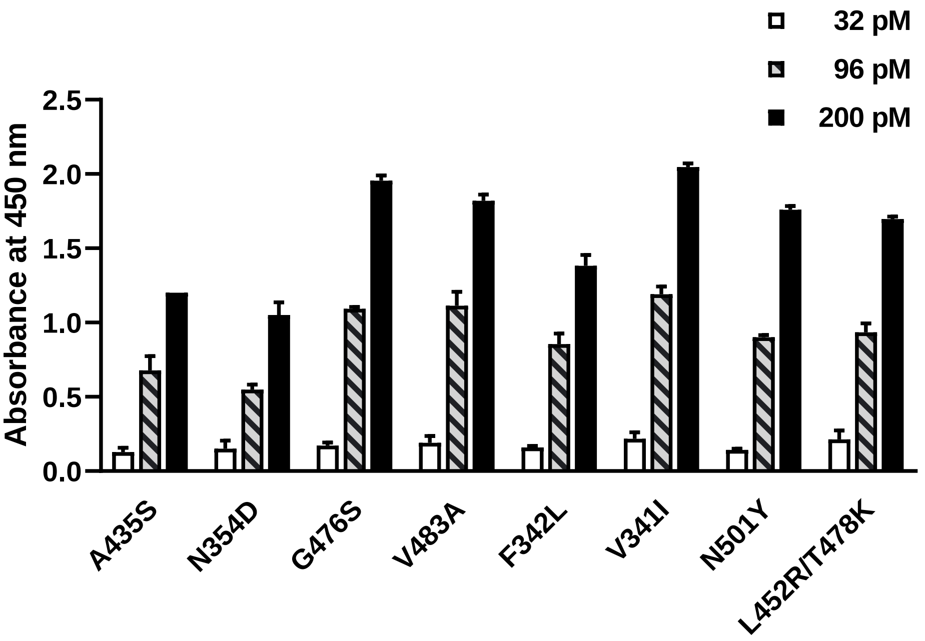

Supplementary figure 2. Detection of multiple SARS-CoV-2 RBD antigens using developed sandwich ELISA. The detection ability of the sandwich ELISA was evaluated against the increasing concentrations of the RBDs of SARS-CoV-2 variants, including A435S, N354D, G476S, V483A, F432L, V341I, N501Y (Alpha), and L454R/T478K (Delta). All values represented as mean ± SD of duplicate measurements and represent one out of two independent experiments.
